# Calculation error following sample dilution: a proposal for processing such specimens using a MUMPS program

**DOI:** 10.1155/S1463924685000219

**Published:** 1985

**Authors:** Thomas G. Pellar, James F. Tuckerman, A. Ralph Henderson

**Affiliations:** Department ofClinical Biochemistry University Hospital (University of Western Ontario) P.O. Box 5339, Postal Stn. A Ontario London N6A 5A5 Canada


Journal of Automatic Chemistry/*Journal of Clinical Laboratory Automation*, Vol. 7, No. 2 (Apr–Jun 1985), pp. 95–98

### Calculation error following sample dilution: a proposal for processing such specimens using a MUMPS program

**Thomas G. Pellar, James F. Tuckerman and A. Ralph Henderson***

*Department of Clinical Biochemistry, University Hospital (University of Western Ontario), P.O. Box 5339, Postal Stn. A, London, Ontario, Canada N6A 5A5*

### Introduction

Experienced laboratory workers recognize that error is an ever-present hazard, and that continual vigilance is the only safeguard, although errors may never be entirely eliminated. In fact, McSwiney and Woodrow [1] have gone as far as to suggest that the incidence may well be irreducible at a level of 2 to 3%; the authors were reminded of this view recently when they discovered a continuing incidence of error associated with the dilution of specimens with analyte concentrations beyond the measurable range of their analysers. On the SMA II (Technicon Instruments Corporation, Tarrytown, New York 10591, USA) and ASTRA 8 (Beckman Instruments, Inc., Brea, California 92621, USA) an actual or expected 'beyond range' specimen is diluted with a pooled laboratory serum containing known, normal, levels of analyte. This technique is also used to 'extend' the volume of a specimen provided in insufficient volume for these analysers. On the enzyme analysers at University Hospital, such as the Mark II Kinetic Analyzer (LKB-Produkter AB, Bromma, Sweden), 'beyond-range' specimens are diluted with 150 mmol/l saline. In all cases, a simple calculation is necessary to obtain the analyte value of the original, undiluted, sample. Unfortunately, for a number of reasons, these simple calculations are not always done correctly, particularly when staff are under pressure. In an initial survey of dilution error (table 1) two types of mistake were discovered—serious (for example an S-urate result of 451 was reported that was actually 801 mol/l), and trivial (for example 762 reported, instead of 774 mol/l, for an S-urate result). As this incidence of error was unacceptable it was decided that a routine should be created, usable by all staff and available on all 27 video terminals of the laboratory computer system, that would allow staff to:

(1) Use a standardized procedure for handling dilutions.
(2) Use the current, updated, values of the serum pool diluent without having to re-enter them.
(3) Produce a record of every such transaction.
(4) Record all dilution calculations on a daily basis as a form of laboratory audit.

* Corresponding author.

### Procedure

On calling the Dilution Routine the operator is asked to select from:

DILUTION CALCULATION/REPORT
ACTIVITY LOG
EDIT POOL VALUES

On selecting the DILUTION CALCULATION/REPORT by entering 'D', the operator is taken through the routine shown in figure 1 (line numbers have been added to facilitate the description of the procedure). The double slashes indicate the default values used if ENTER is pressed. The appropriate diluent is selected (#1) and the specimen identified (#2). On entering T (for today) the date is returned and the sample number (C900) is returned in its correct format – date:C(hemistry)0900. The test mnemonic is entered (#3), and the test name is returned, the units confirmed and the current serum pool identified. The operator is informed when the pool value was last updated (and by whom), and the current analyte concentration; these values can be updated if necessary (#4). The appropriate dilution factor is selected and the value of the diluted result is entered (#5); mistakes can be corrected before printing of the report (#6). If the diluted result is less than, or equal to, the pool result, this is indicated, on the screen, as a warning (also, see below). If a saline dilution is selected on calling the routine the Serum Pool Section is bypassed by the program.

The report (figure 2) is printed using the routine outlined in figure 1. This report is a record of the entire calculation including the identification of the serum pool used for dilution. The technologist is also identified. A warning note:

*'Note: The diluted result is less than, or equal to, the pool result. Please examine all data carefully before using . . .'*

is added when the dilution step has been used to extend the volume of a patient's sample.

The serum pool values (and pool lot number) are updated by means of the EDIT POOL VALUES routine. Analytes are identified by entering the test mnemonic, and the current value (if any) is displayed. Values can be entered or removed, and the routine also checks that the pre-defined test format (see figure 1) is observed, although this check can be overidden. As each analyte value is changed, an entry is automatically made in the

```
                              DILUTION REPORT
----------------------------------------------------------------------------

#1                        SELECT DILUENT USED:

                          1)  Saline
                          2)  Pooled Sera

                          Choice:  2 //

#2  Collection Date:  T // 29 NOV

    Specimen number:  C900  2911:C0900

#3  Diluted test (enter by mnemonic):C   S-Creatinine
    Units for S-Creatinine:  umol/L //
    Lot number of Serum Pool (e.g., 84/25):84/17 //
    S-Creatinine concentration in Serum Pool 84/17 is 155 (29 NOV:PELLAR,T)

#4  Is this correct?  N // Y

#5  Dilution factor (e.g., 1+4 gives a factor of 5):  5
    Specimen 2911:C0900 result at 5-fold dilution for S-Creatinine:301

#6  Is all your input correct?  N // Y
    On output device:  0 // [report is printed - see Figure 2]
    Another dilution to calculate?  N //
```

*Figure 1. The dilution entry. This procedure is password protected so that operator identity is required if the program is to be used. Although not shown, all numbers are format checked (i.e. the actual size of the analyte value is checked against the expected format, for example S-potassium has a format of 1N.1N, S-Sodium 3N.0N, and so on) and the operator has to positively overide this check if the format is unusual. The current pool analyte values are entered either at the time of pool preparation (see text) or during entry of a dilution; this eliminates one source of error identified in the preliminary survey.*

```
                         DEPT OF CLINICAL BIOCHEMISTRY
                         UNIVERSITY HOSPITAL, LONDON

                               DILUTION REPORT

   RUN: 29 NOV 1984                                      TECH: PELLAR,T

        1441

----------------------------------------------------------------------------

                      Specimen number:   2911:C0900

                        Diluted test:   S-Creatinine

               Units for S-Creatinine:   umol/L

               Lot number of Serum Pool:   84/17

         S-Creatinine level in this pool:   155

                      Dilution factor:   5

          Diluted S-Creatinine result :   301

----------------------------------------------------------------------------

                        Specimen number 2911:C0900

                   Calculated S-Creatinine = 885 umol/L

                   ====================================

```

*Figure 2. The Dilution Report. This is printed at the end of the dilution entry (see figure 1). It is filed with the worksheet as the laboratory record of the dilution.*

RUN: 12 DEC 1984 TECH: PELLAR,T

1021

DILUTION REPORT: ACTIVITY LOG
FROM: 11 DEC TO: 11 DEC
PAGE: 1

| DATE | TIME | SPEC# | POOL# | ANALYTE | DILUTED RESULT | CALC. RESULT | DEV | TECH |
|---|---|---|---|---|---|---|---|---|
| 1112 | 0008 | 1012:C0511 | saline | CK | 13 | 65 | 47 | MELANSON,P |
| 1112 | 0605 | 1112:C0011 | saline | CK | 229 | 1145 | 47 | MELANSON,P |
| 1112 | 0627 | 1112:C0018 | saline | GR | 14.2 | 28.4 | 47 | MELANSON,P |
| 1112 | 0749 | 1112:C0024 | saline | CK | 183 | 366 | 47 | THOM,B |
| 1112 | 0945 | 1112:C0999 | 84/19 | CA | 3.20 | 4.00 | 25 | PELLAR,T |
| 1112 | 0947 | 1012:C0354 | 84/19 | UR | 409 | 605 | 48 | FANELLI,I |
| 1112 | 0950 | 1012:C0207 | saline | OT | 19 | 38 | 48 | ROSS,ML |
| 1112 | 1027 | 1112:C0139 | saline | PT | 192 | 576 | 48 | ROSS,ML |
| 1112 | 1049 | 1112:C0999 | 84/19 | CA | 3.20 | 3.84 | 49 | PELLAR,T |
| 1112 | 1050 | 1112:C0999 | saline | OT | 32 | 320 | 19 | PELLAR,T |
| 1112 | 1202 | 1112:C0034 | 84/19 | BIT | 77.7 | 212.9 | 47 | DAVIS,J |
| 1112 | 1304 | 1112:C0296 | saline | CK | 299 | 897 | 48 | BROOKS,K |
| 1112 | 1414 | 1012:C0449 | saline | AMY | 4 | 8 | 48 | DOHERTY,K |
| 1112 | 1459 | 1112:C0077 | 84/19 | UR | 584 | 1132 | 47 | ALBANO,J |
| 1112 | 1500 | 1112:C0077 | 84/19 | PR | 60.5 | 62.5 | 47 | ALBANO,J |
| 1112 | 1501 | 1112:C0086 | 84/19 | UR | 460 | 760 | 47 | ALBANO,J |
| 1112 | 1539 | 1112:C0076 | saline | GGT | 200 | 400 | 48 | ROSS,ML |
| 1112 | 1539 | 1112:C0080 | saline | GGT | 231 | 693 | 48 | ROSS,ML |
| 1112 | 1930 | 1112:C0127 | saline | U | 33.2 | 66.4 | 47 | ESBJERG,A |
| 1112 | 1930 | 1112:C0429 | saline | GR | 15.0 | 30.0 | 47 | ESBJERG,A |
| 1112 | 1943 | 1112:C0245 | 84/19 | BIT | 7.7 | 2.9 | 47 | SMITH,D |
| 1112 | 1944 | 1112:C0245 | 84/19 | PR | 65.9 | 78.7 | 47 | SMITH,D |
| 1112 | 1944 | 1112:C0286 | 84/19 | BIT | 80.0 | 219.8 | 47 | SMITH,D |
| 1112 | 2109 | 1112:C0477 | saline | CK | 225 | 1125 | 47 | ESBJERG,A |
| 1112 | 2159 | 1112:C0370 | 84/19 | U | 17.8 | 38.2 | 47 | SMITH,D |

*Figure 3. The Dilution Activity Log. A segment of this report is shown, which contains sufficient information for the calculations to be repeated using the pool # analyte value (see figure 4). A saline dilution is reported as 'saline'. The date is the leading entry in the field to allow rapid deletion of log entries, by day, once they have been printed.*

RUN: 12 DEC 1984 TECH: PELLAR,T

1032

DILUTION REPORT: POOL EDIT ACTIVITY LOG
FROM: 12 DEC TO: 12 DEC
PAGE: 1

| DATE | TIME | TEST | OLD RESULT | NEW RESULT | TECH | |
|---|---|---|---|---|---|---|
| 12 DEC 1984 | 0949 | A | 36.9 | 40.1 | DAVIS,J | |
| 12 DEC 1984 | 0950 | CA | 2.19 | 2.16 | DAVIS,J | |
| 12 DEC 1984 | 0950 | U | 7.6 | 7.7 | DAVIS,J | |
| 12 DEC 1984 | 0950 | PR | 59.5 | 60.5 | DAVIS,J | |
| 12 DEC 1984 | 0950 | PO | 1.14 | 1.13 | DAVIS,J | |
| 12 DEC 1984 | 0950 | UR | 310 | 309 | DAVIS,J | |
| 12 DEC 1984 | 1029 | BIT | | | PELLAR,T | AUTOKILL |
| 12 DEC 1984 | 1029 | BIT | | 10.1 | PELLAR,T | |

*Figure 4. The Edit Pool Activity Log. This report is printed with the dilution activity log (figure 3). It records all changes in the pool analyte values and the date and time of change, together with he name of the technologist making these changes.*

POOL EDIT ACTIVITY LOG listing the time and date of change, the old and new values and the name of the person making the change.

If the date of a pool is not 'today' then the pool values are automatically deleted (AUTOKILL – see figure 4) thus forcing daily review of all pool values. As previously mentioned, the DILUTION CALCULATION/ REPORT routine also allows pool values (and lot number) to be updated(see figure 1). These updates are also logged. In figure 4 we show a portion of the POOL EDIT ACTIVITY LOG; it will be noted that AUTO-KILL was invoked when a calculation was called that required yesterday's (i.e. 11 December) pool value for BIT (total bilirubin), thus forcing the entry of the current (i.e. 12 December) value.

Finally, for the purposes of laboratory audit, we print a daily ACTIVITY LOG of each dilution transaction and the associated POOL EDIT ACTIVITY LOG. A section of these logs are shown in figures 3 and 4. The existence of these documents allows the laboratory supervisor to review all dilutions done during each shift. Errors of program misuse are therefore readily identified and corrective re-training instituted.

### Discussion

It was previously assumed that the procedure for handling dilutions at University Hospital worked satisfactorily. A second technologist always checked these calculations. However, the discovered incidence of error (table 1) indicated that errors (arithmetical or using the incorrect pool value) were occurring too frequently. The revised procedure has totally eliminated this source of error (table 1), although it is intended that long-term spot checking will be set up to ascertain the continuing effectiveness of this new routine. Perhaps part of the success of this arrangement lies in the ready availability of the dilution programme on *any* terminal: the technologist does not have to use a special microcomputer. The programme was rapidly accepted, and used, by all laboratory staff with little hesitation (a good measure of the utility of a new procedure).

*Table 1. Incidence of error in 100 sequential dilutions.*

| Error* | Initial survey (May 1984) | Post-implementation survey (June 1984) | Continuing surveys (July, September and November 1984) |
|---|---|---|---|
| Serious error | 8% | Nil | Nil |
| Trivial error | 4% | Nil | Nil |
| Total error | 12% | Nil | Nil |

* Serious error indicates a significant difference between the actual and reported value. Trivial error indicates a difference of no consequence. Actual values were calculated independently by a third party.

The authors expect that their discovered error rate in the handling of dilutions is not unique. This Department is staffed 24 h each day and more than two million tests are analysed annually. Most teaching hospitals in North America handle this type of work-load, so dilutional errors are probably more frequent than is generally realized. Interestingly, McSwiney and Woodrow [1] found that 6% of all their detected errors were due to miscalculation. They observed that these errors could not be eliminated by exhortation or example, and the best solution to date has been forms designed so that a series of simple stages leads to the correct results. The interactive routine used at University Hospital is clearly analogous to the series of simple stages used by McSwiney and Woodrow.

This type of study is a legitimate aspect of laboratory quality assurance. This is usually judged by performance in external quality-control schemes [2,3 and 4], although there is an increasing realization that many other laboratory functions can, and should be, assessed [3 and 5]. A feature of the system described is the careful tracking of the technologist doing the dilutions and setting the pool values; this allows easy audit of the effectiveness of procedures – an important aspect of an efficient quality assurance system.

A copy of this program, written in the MIIS dialect of MUMPS, and using the appropriate global files from University Hospital's MEDITECH laboratory computer system is available from Dr Henderson. Enough detail is supplied to allow a user of a MUMPS system to create their own globals for the user, test and other files.

### References

1. McSwiney, R. R. and Woodrow, D. A., *Journal of Medical Laboratory Technology*, **26** (1969), 340.
2. Whitehead, T., in *A Question of Quality?: Roads to Assurance in Medical Care*, Ed. McLaughlan, G. (Oxford University Press, London, 1976), p. 97.
3. Baron, D. N., in *Reviewing Practice in Medical Care: Steps to Quality Assurance*, Ed. McLaughlan G. (Nuffield Provincial Hospitals Trust, London, 1981), p. 57.
4. Maxwell, R. J., *British Medical Journal*, **288** (1984), 1470.
5. Anon., *Lancet*, **ii** (1982), 196.

